# Synthesis and Antimicrobial Activity of δ-Viniferin Analogues and Isosteres

**DOI:** 10.3390/molecules26247594

**Published:** 2021-12-15

**Authors:** Luce Micaela Mattio, Cecilia Pinna, Giorgia Catinella, Loana Musso, Kasandra Juliet Pedersen, Karen Angeliki Krogfelt, Sabrina Dallavalle, Andrea Pinto

**Affiliations:** 1 Department of Food, Environmental and Nutritional Sciences (DeFENS), University of Milan, Via Celoria 2, 20133 Milan, Italy; luce.mattio@unimi.it (L.M.M.); cecilia.pinna@unimi.it (C.P.); giorgia.catinella@unimi.it (G.C.); loana.musso@unimi.it (L.M.); andrea.pinto@unimi.it (A.P.); 2 Institute of Molecular and Medical Biology, Roskilde University, 4000 Roskilde, Denmark; kjuliet@ruc.dk (K.J.P.); karenak@ruc.dk (K.A.K.)

**Keywords:** viniferin derivatives, stilbenoids, benzofuran nucleus, antimicrobials

## Abstract

The natural stilbenoid dehydro-δ-viniferin, containing a benzofuran core, has been recently identified as a promising antimicrobial agent. To define the structural elements relevant to its activity, we modified the styryl moiety, appended at C5 of the benzofuran ring. In this paper, we report the construction of stilbenoid-derived 2,3-diaryl-5-substituted benzofurans, which allowed us to prepare a focused collection of dehydro-δ-viniferin analogues. The antimicrobial activity of the synthesized compounds was evaluated against *S. aureus* ATCC29213. The simplified analogue 5,5′-(2-(4-hydroxyphenyl)benzofuran-3,5-diyl)bis(benzene-1,3-diol), obtained in three steps from 4-bromo-2-iodophenol (63% overall yield), emerged as a promising candidate for further investigation (MIC = 4 µg/mL).

## 1. Introduction

Resveratrol-derived natural products, belonging to the class of polyphenolic stilbenes, have increasingly attracted the attention of the scientific community because of their diverse biological activities and intriguing molecular architectures [1,2,3]. Nonetheless, the growing interest in the pharmacological potential of this class of molecules derives from the poor understanding of the in vivo mechanisms of action of their parent compound resveratrol, which severely limits its therapeutic use [4] and the necessity to increase its low bioavailability and in vivo stability. Over the last years, several efforts were made towards the synthesis of complex natural resveratrol oligomers, by biomimetic and de novo approaches [1,5,6,7,8,9]. However, only few research groups have focused on the synthesis of new resveratrol-derived chemical scaffolds with improved pharmacodynamics and pharmacokinetics with respect to the natural precursors [6,10,11,12,13,14]. In this scenario, we planned to set up a versatile and efficient synthetic strategy for the construction of dimeric resveratrol-derived benzofurans. Benzo[b]furan-containing molecules, present in numerous bioactive natural compounds, have been extensively studied because of their wide array of biological activities, including anticancer, antimicrobial, immunomodulatory, antioxidant, and anti-inflammatory properties [15,16,17,18]. It is noteworthy that, in the last years, the benzofuran motif has been revealed to be a pharmacophore of choice for the design of new antimicrobial agents [19,20]. We have recently reported the synthesis and the antimicrobial activity evaluation of a collection of resveratrol-derived monomers (i.e., resveratrol, pterostilbene, and piceatannol) and dimers (i.e., trans-δ-viniferin, trans-ε-viniferin, pallidol, dehydro-δ-viniferin, and viniferifuran) against a series of foodborne pathogens [21].

## 2. Results and Discussion

**Figure 1:**
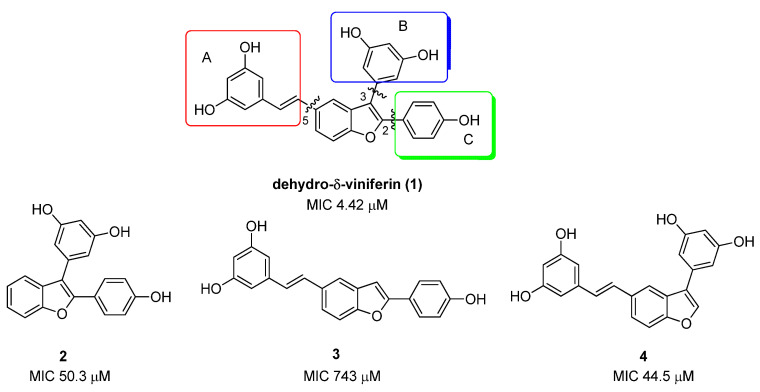
Structures and MIC values of model compounds **2**, **3** and **4**.

Dehydro-δ-viniferin (**1**, Figure 1), containing a benzofuran core, was identified as a promising compound against Gram-positive bacteria. In particular, it was shown to exert its antimicrobial activity on the foodborne pathogen *Listeria monocytogenes* Scott A, used as model of Gram-positive microorganisms (MIC and MBC values of 4.42 and 35.3 µM, respectively) [21]. The compound causes significant cytoplasmic membrane damage, by membrane depolarization, loss of membrane integrity, and severe morphological changes.

A previous SAR study performed by our group on simplified analogues of **1** (compounds **2**, **3**, **4**) [22], which were obtained by the selective removal of the moieties linked in positions two, three, and five of the benzofuran core, showed that none of the structurally simplified compounds resulted to be more active than the precursor (Figure 1). In particular, a drastic drop of the antibacterial activity, due to the fatal lack of ring B, was observed for the derivative **3** (MIC value of 743 µM against 4.42 µM of dehydro-δ-viniferin), thus suggesting the fundamental role of the aryl ring in position three of the benzofuran core. An important loss of antimicrobial activity, albeit to a lesser extent, was observed for compounds **2** and **4**, obtained by the removal of the styryl group at position five and of the aryl ring in position two, respectively (MIC values of 50.3 µM (**2**) and 44.5 µM (**4**), vs. 4.42 µM (**1**)) (Figure 1).

Thus, we planned to prepare a novel set of dehydro-δ-viniferin analogues and isosteres, obtained by modifying the styryl moiety A (Figure 1), while maintaining the unaltered rings B and C. In particular, a removal of the double bond or its replacement with moieties such as an amide, alkyne or a saturated chain, could clarify the role of the geometry and stereoelectronic effects for the antimicrobial activity. In addition, we planned to synthesize dehydro-δ-viniferin analogues that maintained the stilbene double bond, having, however, aromatic rings that were different from the resorcinol moiety.

**Figure 2:**
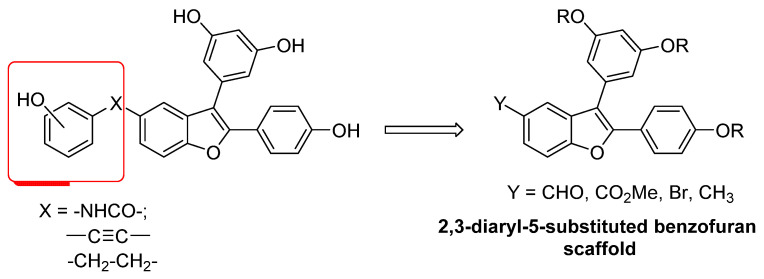
Retrosynthetic analysis for the obtainment of desired compounds.

In this perspective, we needed a versatile strategy to construct the 2,3-diaryl benzofuran ring bearing on C-5 a proper functional group (X) for the insertion of the appropriate fragment (Figure 2).

Among the various methods to access stilbenoid-derived 2,3-diaryl-5-substituted benzofurans [23,24,25,26,27,28], palladium catalysed reactions have proven to be rapid and convenient. In particular, an efficient one-pot method developed by Cacchi and coworkers [29] and successively implemented by Markina and coworkers [30], involves a Sonogashira coupling between an *ortho*-iodophenol and an aryl-substituted terminal alkyne to generate, at room temperature, the corresponding internal alkyne. The alkynylphenol obtained as an intermediate undergoes a simultaneous cyclization with the adjacent phenol group and an oxidative addition with the aryl-iodide-palladium complex with CuI, in acetonitrile at 100 °C, under microwave irradiation. Using this approach, we obtained C5-substituted 2,3-diarylbenzofurans in a three-component one-pot reaction in 48–72% yields.

**Scheme 1:**
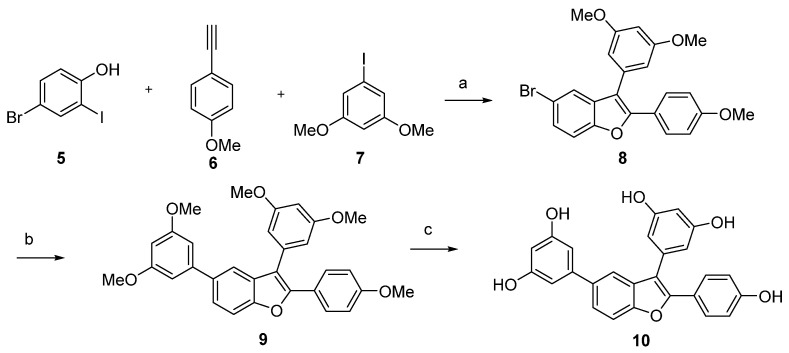
*Reagents and conditions*: (**a**) (i) PdCl_2_(PPh_3_)_2_∙DCM, CuI, THF/TEA 1:3, rt, MW, 30 min, (ii) ACN, 100 °C, MW, 25 min, 72%; (**b**) (3,5-dimethoxyphenyl)boronic acid, Pd(PPh_3_)_4_, DMF/EtOH 1:1, aq 1 M Cs_s_CO_3_, 120 °C, 20 min, MW, 91%; (**c**) BBr_3_ 1 M DCM, DCM, −78 °C to rt, overnight, 96%.

Specifically, we generated the bromo functionalized intermediate **8** by reaction of 4-bromo-2-iodophenol **5**, 4-ethynylanisole **6** and 3,5-dimethoxy-1-iodobenzene **7** (Scheme 1). Compound **8** underwent a Suzuki-coupling with (3,5-dimethoxyphenyl)boronic acid with Pd(PPh_3_)_4_ and aqueous 1 M Cs_2_CO_3_ in a mixture DMF/EtOH (1:1), under microwave irradiation, for 20 min at 120 °C [30] to afford compound **9** in 91% yield. Final demethylation with BBr_3_ provided **10**, as a simplified analogue of our hit compound **1**, lacking the stilbene double bond.

**Scheme 2:**
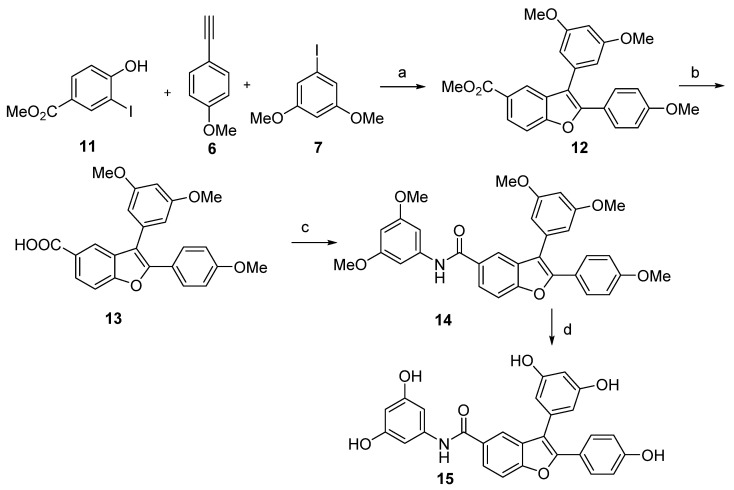
*Reagents and conditions*: (**a**) (i) PdCl_2_(PPh_3_)_2_∙DCM, CuI, THF/TEA 1:3, rt, MW, 30 min, (ii) ACN, 100 °C, MW, 25 min, 66%; (**b**) LiOH∙H_2_O, THF/H_2_O 1:1, rt, 24 h, quantitative yield; (**c**) (i) EDC∙HCl, HOBt, DMF; 0 °C to rt, 90 min, (ii) 3,5-dimethoxyaniline, DIPEA, DMF, 0 °C to rt, overnight, 70%; (**d**) BBr_3_ 1 M DCM, DCM, −78 °C to rt, overnight, 73%.

Then, we focused on the synthesis of the isosteres bearing an amide in place of the double bond. Amide isosteres of resveratrol have shown activity similar to the parent compound [31]. The amide linkage should allow to maintain the transoid architecture of the *trans*-stilbene, conferring however improved solubility and increased polarity [32,33] as well as differences in electronic perturbations [32,33]. Therefore, analogue **15** was synthesized (Scheme 2). The Sonogashira/Cacchi type cyclization of the commercially available methyl 4-hydroxy-3-iodobenzoate **11**, 4-ethynylanisole **6** and 3,5-dimethoxy-1-iodobenze **7** gave the desired benzofuran **12** in 66% yield. Hydrolysis of the ester **12** was performed with LiOH∙H_2_O in a mixture of THF/water 1:1 for 24 h. The resulting carboxylic acid **13** was reacted with 3,5-dimethoxyaniline, in presence of EDC∙HCl and HOBt, to give amide **14**, which was demethylated with BBr_3_ to afford compound **15** in 73% yield.

**Scheme 3:**
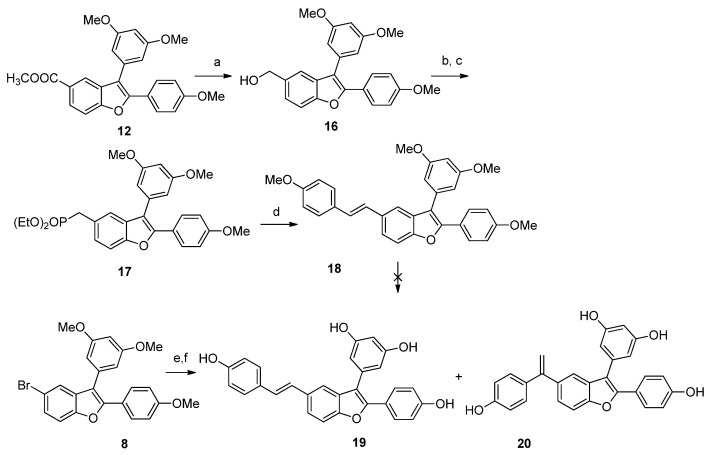
*Reagents and conditions*: (**a**) LiAlH_4_, THF, 0 °C, 20 min, quantitative yield; (**b**) PBr_3_, cat pyridine, Et_2_O, rt to reflux, 2 h, (**c**) P(OEt)_3_, 130 °C, overnight, 80% over two steps; (**d**) 4-methoxybenzaldehyde, NaH, 120 °C, 30 min, MW, 86%; (**e**) BBr_3_ 1 M DCM, DCM, −78 °C to rt, overnight, 87%; (**f**) 4-hydroxystirene, TEA, dppp (1,3-bis(diphenylphosphino)propane), Pd(OAc)_2_, dry DMF, 120 °C, 48 h.

The ester **12** was envisaged as a versatile intermediate for the preparation of a set of dehydro-δ-viniferin derivatives, differently substituted on ring A (Scheme 3). Reduction with LiAlH_4_ gave, quantitatively, compound **16**, which was converted into the corresponding bromide derivative with PBr_3_. Reaction with triethyl phosphite at 130 °C overnight, which afforded the phosphonate **17** in 80% yield over two steps. The HWE reaction with 4-methoxybenzaldehyde provided the desired stilbene **18**, only as a *trans* isomer, in 86% yield. Unfortunately, attempts to deprotect the methyl groups with BBr_3_ at −78 °C in dry DCM, following the usual procedure, gave only degradation products.

Several troublesome efforts in the demethylation process confirmed that this step is an Achilles’ heel in the synthesis of stilbenoids-derived compounds [6,10,14,22].

Methyl groups are convenient protecting groups for phenolic moieties because of the availability of their starting reagents and their high stability to a wide variety of reaction conditions. However, as a not-negligible drawback, their high robustness requires harsh conditions in the deprotection step, often resulting in poor yields and product degradation in the presence of highly reactive double bonds [5,6,10,22].

As stilbenoids are known to form dimers and polymers with a variety of acids, including BBr_3_ [34,35], alternative protocols were investigated. We first attempted to obtain the desired compound **19** by the initial deprotection of bromoderivative **8**, followed by a direct insertion of the *p*-hydroxystyryl moiety via the Heck reaction. However, the reaction gave a mixture of **19** and its isomer **20**, coeluted in column chromatography (Scheme 3).

**Scheme 4:**
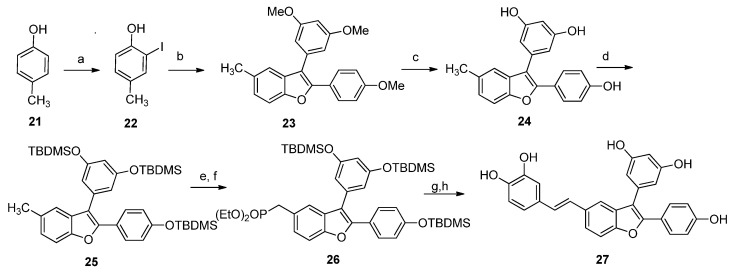
*Reagents and conditions*: (**a**) (i) p-TsOH∙H_2_O, ACN, rt, 10 min; (ii) NIS, rt, overnight, 97%; (**b**) (i) 4-ethynianisole, PdCl_2_(PPh_3_)_2_∙DCM, CuI, THF/TEA 1:3, rt, MW, 30 min, (ii) 3,5-dimethoxy-1-iodobenzene, ACN, 100 °C, MW, 25 min, 48%; (**c**) BBr_3_ 1 M DCM, DCM, −78 °C to rt, overnight, 90%; (**d**) TBDMSCl, imidazole, DCE, 60 °C, 8 h, 86%; (**e**) NBS, AIBN, CCl_4_, reflux, 8 h, 37%; (**f**) P(OEt)_3_, 130 °C, overnight, 84%; (**g**) 4-bis((*tert*-butyldimethylsilyl)oxy)benzaldehyde, NaH, THF, 0 °C to rt, 24 h, 52%; (**h**) TBAF, THF, 0 °C to rt, 2 h, 60%.

In another synthetic route, 2-iodo-4-methylphenol **22**, prepared in excellent yields from *para*-cresol (**21**) with *N*-iodosuccinimide and *para*-toluenesulfonic acid in acetonitrile [36], was used as the starting material (Scheme 4). In the one-pot-Sonogashira-Cacchi reaction conditions, the obtained intermediate gave the desired benzofuran derivative **23** in 48% yield. Intermediate **23** was smoothly demethylated to afford compound **24** in 90% yield. The protection of hydroxy groups with *tert*-butyldimethylsilylchloride and imidazole was performed in 1,2-dichloroethane at 60 °C, to give compound **25** in a good yield (86%) [8]. Then, a radical bromination of the methyl group with NBS and AIBN as a radical initiator at reflux in CCl_4_ gave a brominated intermediate, which was converted into the corresponding phosphonate **26** with triethyl phosphite at 130 °C (84% yield). The intermediate **26** was reacted with the properly protected 3,4-bis((*tert*-butyldimethylsilyl)oxy)benzaldehyde in presence of LDA in THF in 16% yield. The use of NaH increased the yield to 52%. Eventually, the deprotection of silyl groups was performed with tetrabutylammonium fluoride (TBAF) at 0 °C in THF, to afford compound **28** with a catechol on the styryl moiety (60% yield).

**Scheme 5:**
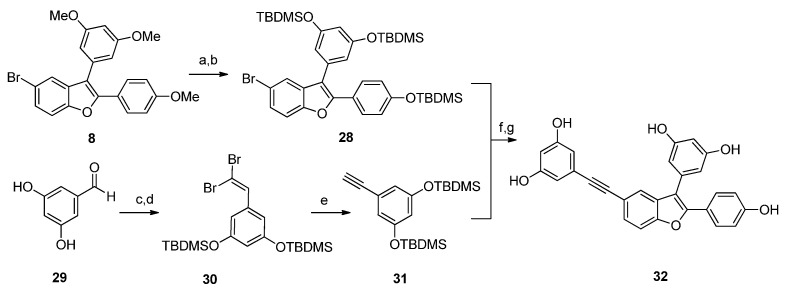
*Reagents and conditions*: (**a**) BBr_3_ 1 M DCM, dry DCM, −78 °C to rt, overnight, 87%; (**b**) TBDMSCl, imidazole, DMF, 0 °C to rt, overnight, 81%; (**c**) TBDMSCl, imidazole, DMF, 0 °C to rt, overnight, 74%; (**d**) CBr_4_, PPh_3_, DCM, 0 °C to rt, 1 h, 83%, (**e**) LDA, THF, −78 °C, 1 h, 91%; (**f**) Pd(PPh_3_)_4_, CuI, TEA, 90 °C, 8 h; (**g**) KF, MeOH/THF, rt, overnight, 38% over 2 steps.

The protection of phenol groups as *t*-butyldimethylsilylethers was applied also to the synthesis of the alkyne derivative **32** (Scheme 5). The high-yield demethylation of brominated intermediate **8** was thus followed by protection of the hydroxy groups as *tert*-butyldimethylsilyl ethers (**28**).

The alkyne **31** was obtained starting from 3,5-dihydroxybenzaldehyde **29**, which was properly protected and then subjected to Corey-Fuchs conditions [37] to give the terminal dibromoalkene **30**, which underwent lithium-halogen exchange and α-elimination with LDA to afford **31** in excellent yield.

The final Sonogashira coupling was performed with Pd(PPh_3_)_4_ and CuI in triethylamine at reflux for 8 h. The crude compound obtained was directly deprotected with KF to give the desired alkyne **32** in 38% yield, over two steps.

**Scheme 6:**
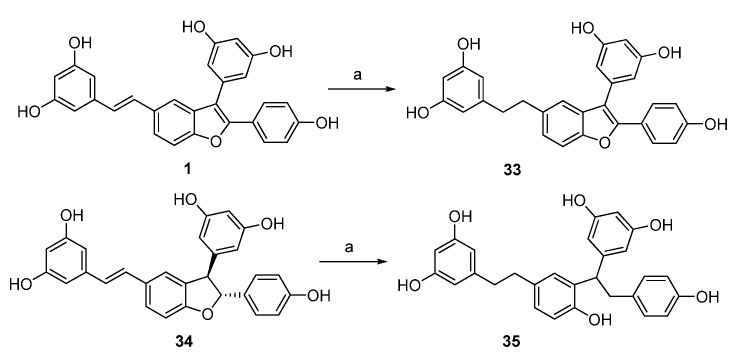
*Reagents and conditions*: (**a**) H_2_/Pd/C 10% wt, EtOH, rt, 4 h, quantitative yield.

Finally, compound **33**, having a saturated chain in place of the stilbene double bond, was obtained in a quantitative yield by the hydrogenation of dehydro-δ-viniferin **1** with Pd/C in ethanol at room temperature for 3 h (Scheme 6). Hydrogenation of δ-viniferin **34**, applying the same protocol, led to a dihydrobenzofuran ring cleavage (compound **35**) [38].

The model compound **1** and the novel derivatives **10**, **15**, **27**, **32**, **33**, and **35** were tested against *S. aureus* ATCC29213, and the minimum inhibition concentration (MIC) and minimal bactericidal concentration (MBC) were determined. The concentration range was 0.25–512 µg/mL for the synthesized compounds and 0.5–64 µg/mL for the reference compound tobramycin. The results are reported in Table 1. The MIC was evaluated using two different growth media: Mueller Hilton Broth, cation adjusted (MHB-II 212322, Becton Dickinson and Company, 7 Loveton Circle Spark, MD, USA), and Tryptic Soy Broth (22092-500 G, MERCK, Vandtårnsvej 62A, 5 sal. Søborg, Denmark).

It has been shown that the growth media play an important role in the outcome of bacterial susceptibility to different charged peptides. Antimicrobial assays were performed in MHB cation-adjusted medium, a complex growth medium [39], and also in the less complex medium TSB [21]. In TSB we achieved approximately equal susceptibility results, uniform growth, and less variation in the repeated independent experiments. Unexpectedly, in both sets of experiments we noticed that at high concentrations the active compounds lost their ability to inhibit the growth of the microorganism. In particular, in the MHB-II medium, compound **1** lost its activity at concentrations higher than 8 µg/mL, compounds **10**, **15**, **27**, and **32** at concentrations higher than 32 µg/mL, and compound **33** at concentrations higher than 16 µg/mL. A similar behaviour for all the compounds was observed in the TSB medium. These results could be explained, considering a self-aggregation process of the tested compounds in the solvent system.

In the MHB-II medium, the MICs of tested compounds ranged from 2 to 256 µg/mL. The majority of compounds showed detectable antimicrobial activity in the MIC range of 2–16 µg/mL. The removal of the double bond (compound **10**; MIC 4 µg/mL), as well as the reduction of the double bond (compounds **33**; MIC 2 µg/mL) and the replacement with the triple bond (compound **32**; MIC 4 µg/mL), gave compounds which maintained a significant activity. Conversely, the replacement of the double bond with an amide group (compound **15**) was deleterious (MIC 16 µg/mL). Also, the replacement of ring A with a catechol was not successful in terms of activity, as compound **27** had a MIC of 16 µg/mL. Compound **35**, obtained by opening the benzofuran system, showed a very high MIC (256 µg/mL). This result confirmed that the heterocyclic ring plays an essential role for antimicrobial activity.

## 3. Materials and Methods

Synthesis. All chemicals used were of analytical grade. Procedures for the synthesis and characterization data for the various derivatives and intermediates are detailed in the Supplementary Materials.

Determination of minimum inhibition concentration (MIC) and minimum bactericidal concentration (MBC). The minimum inhibition concentration (MIC) of compounds was determined for *S. aureus* ATCC29213. The concentration range of the compounds were 0.25–512 µg/mL. Tobramycin (T2503, TCI Europe N.V) was used as a control with a concentration range of 0.5–64 µg/mL. One colony of *S. aureus* was inoculated in 5 mL growth media and incubated overnight in a water bath at 37 °C, 180 rmp. Three biological replicas were used. The overnight cultures were diluted 1:50 and grown to exponential phase at OD600~0.4, either in MHB-II and in TSB. The bacterial culture was diluted 1:500 and transferred to a microdilution plate together with the compounds. The plate was then sealed and incubated overnight at 37 °C. After incubation, the plates were examined for microbial growth. A CFU assay was performed to estimate the final concentration of the 1:500 diluted culture. The expected concentration range was 2 × 10^5^–8 × 10^5^ CFU/mL. The results were obtained 24 h after incubation. To determine the MBC, 10 µL of each compound concentration from the MIC, was transferred to LB (L3022 Sigma Aldrich) agar plates. The plates were incubated overnight at 37 °C. After incubation, the concentration at which no visible microbial growth was found was considered as the MBC.

## 4. Conclusions

The resveratrol dimer dehydro-δ-viniferin, containing a benzofuran core, has been identified as a promising antimicrobial compound. As part of the research for new antimicrobials, our recent interest has been directed to the synthesis of new dehydro-δ-viniferin analogues, to gain insights into the structural determinants for their activity. We investigated various protocols to access stilbenoid-derived 2,3-diaryl-5-substituted benzofurans, evidencing critical steps such as the demethylation of phenolic groups. Following these strategies, we prepared a focused collection of analogues, which were tested to evaluate their antimicrobial activity. Because of the modular nature of the synthetic approaches, ready access to diversity-oriented libraries of stilbenoid derived-benzofurans could be available.

Our study has evidenced that the styryl moiety, appended at C5 of the benzofuran ring, can be modified without affecting the antimicrobial activity of the compounds. Notably, the removal of the double bond (compound **10**) andits conversion into a rigid linear triple bond (compound **32**), or into a more flexible saturated chain (compound **33**), gave compounds which were still endowed with significant antimicrobial activity. In this context, the simplified analogue **10** could represent a promising model compound for further development and investigation.

## Figures and Tables

**Table 1:** Susceptibility testing of *S. aureus* towards model compound **1** and newly synthesized analogues.

		*S. aureus* ATCC29213			
		MHB-II		TSB	
Compounds	Structure	MIC (µg/mL)	MBC (µg/mL)	MIC (µg/mL)	MBC (µg/mL)
**1**	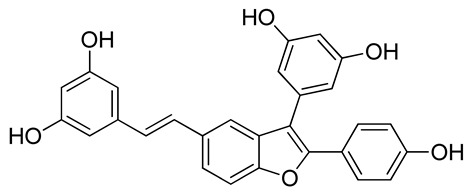	2 *	≥512	≥512	≥512
**10**	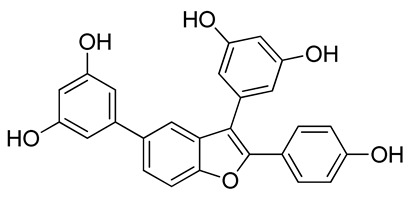	4 *	≥512	32 *	≥512
**15**	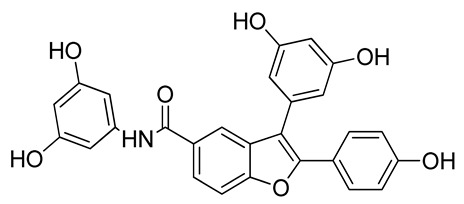	16 *	≥512	32 *	≥512
**27**	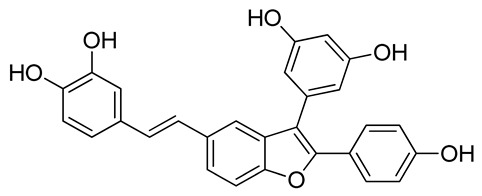	16 *	≥512	16 *	16 *
**32**	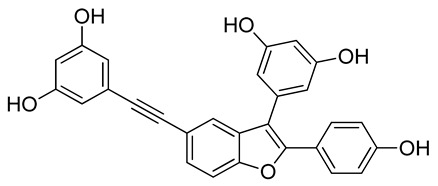	2 *	8	2 *	2 *
**33**	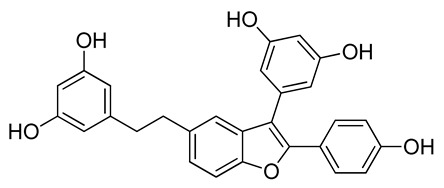	4 *	4	4 *	4 *
**35**	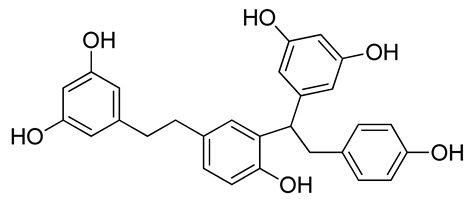	256	≥512	≥512	≥512
**Tobramycin**		<0.5	<0.5	<0.5	<0.5

* The bacteria were able to grow at high concentrations of the tested compounds (see main text).

## Data Availability

Not applicable.

## Supplementary Materials

The following are available online. Synthesis and characterization of compounds **1**, **10**, **15**, **27**, **32**, **33**, **35** [40,41].

## Sample Availability

Samples of the compounds **1**, **10**, **15**, **27**, **32**, **33**, **35** are available from the authors.
