# Supplementary material for: Synthesis and Antimicrobial Activity of δ-Viniferin Analogues and Isosteres
Source: Molecules. 2021 Dec 15;26(24):7594. doi: 10.3390/molecules26247594 (PMC8703454; doi:10.3390/molecules26247594)
Supplement: Supplementary file 1 [file molecules-26-07594-s001.zip › molecules-1479046-supplementary.pdf]

Synthesis and antimicrobial activity of δ-viniferin analogues and isosteres

Luce Micaela Mattio, Cecilia Pinna, Giorgia Catinella, Loana Musso, Kasandra Juliet Pedersen, Karen Angeliki Krogfelt, Sabrina Dallavalle and Andrea Pinto.

Materials and methods. General information. S1

Synthesis and characterization of tested compounds. S2-S13

Materials and methods. General information.

All reagents and solvents were of reagent grade or were purified by standard methods before use. All reactions requiring anhydrous conditions were performed under nitrogen or argon atmosphere with Schlenk-type oven-dried and/or flame-dried glassware.

Melting points were determined on a model B-540 Büchi apparatus and are uncorrected. NMR data were acquired using a Varian Mercury-300 MHz spectrometer (Varian, Palo Alto, CA, USA), Bruker AV600. Chemical shifts (δ values) and coupling constants (*J* values) are given in ppm and Hz, respectively. The elemental analyses were recorded on a CARLO ERBA EA 1108 instrument. Isolation and purification of the compounds were performed by flash column chromatography on silica gel 60 (230–400 mesh). The analytical thin-layer chromatography (TLC) was conducted on TLC plates (silica gel 60 F254, aluminium foil). Substances were detected with a UV-light source (λ = 254 or 365 nm) or stained with 10% phosphomolybdic acid solution (10.0 g phosphomolybdic acid in 100 mL of abs. ethanol), or with ninhydrin solution (1.5 g ninhydrin, 3.0 mL acetic acid in 100 mL of abs. ethanol), or with 2,4-dinitrophenylhydrazine solution (12 g 2,4-dinitrophenylhydrazine, 60 mL conc. sulfuric acid, 80 mL water in 200 mL of 95% ethanol), or with potassium permanganate solution (1.5 g $KMnO_4$, 10 g K_2CO_3, 1.25 mL 10% NaOH in 200 mL of water).

5-Bromo-3-(3,5-dimethoxyphenyl)-2-(4-methoxyphenyl)benzofuran (8). 4-bromo-2-iodophenol **5** (1g, 3.411 mmol, 1 eq) and $PdCl_2(PPh_3)_2$ (72 mg, 0.102 mmol, 0.03 eq) were dissolved in dry THF (3.4 mL) in a microwave vial under N_2 atmosphere. Dry TEA (10.2 mL) and CuI (13 mg, 0.068 mmol, 0.02 eq) were added and the reaction mixture was stirred for 10 min. Then, **6** (530 µL, 4.09 mmol, 1.2 eq) was added and the mixture was stirred in a microwave reactor for 30 min at room temperature. After that, 1-iodo-3,5-dimethoxybenzene **7** (900 mg, 3.411 mmol, 1 eq) and dry acetonitrile (13.6 mL) were added and the mixture was stirred at 100 °C for 25 min, under microwave irradiation. The reaction mixture was allowed to cool to room temperature and the solvent was evaporated. The crude was purified on silica gel using as eluent CHX/DCM (from 65:35 to 6:4) to give the desired compound as a yellowish solid (72% yield). M.p.: 125-126°C. R_f: 0.5 (CHX/DCM 6:4).^{1}H NMR (300 MHz, CD_3OD) δ (ppm): 7.65 – 7.58 (m, 3H), 7.44 – 7.39 (m, 2H), 6.90 – 6.84 (m, 2H), 6.60 (d, J = 1.9 Hz, 2H), 6.52 (t, J = 1.9 Hz, 1H), 3.83 (s, 3H), 3.79 (s, 3H). ^{13}C NMR (150 MHz, CD_3OD) δ (ppm): 161.3 (×2C), 160.0, 152.4, 152.0, 134.2, 132.4, 128.6 (×2C), 127.0, 122.6, 122.4, 116.0, 115.4, 113.9 (×2C), 112.4, 107.6 (×2C), 100.0, 55.4, 55.3 (×2C).

3,5-Bis(3,5-dimethoxyphenyl)-2-(4-methoxyphenyl)benzofuran (9). In a microwave vial, compound **8** (40 mg, 0.091 mmol, 1 eq) and 3,5-dimethoxyphenylboronic acid (0.109 mmol, 20 mg, 1.2 eq) were dissolved in a mixture DMF/EtOH 1:1 (1.5 mL), previously degassed, under N_2 atmosphere. Then, $Pd(PPh_3)_4$ (5.3 mg, 0.0045 mmol, 0.05 eq) and aq 1M Cs_2CO_3 (0.23 mL, 0.230 mmol, 2.5 eq) were added and the resulting mixture was stirred in at 120 °C for 20 min under microwave irradiation. The mixture was cooled, diluted with EtOAc, and washed three times with a mixture water/brine 1:1. The organic layer was dried over anhydrous Na_2SO_4, filtered, and evaporated. The crude product was purified on silica gel by column chromatography using CHX/AcOEt 9:1 as eluent. The product was obtained as a white solid (91%) [30]. M.p.: 105 – 107 °C. R_f: 0.24 CHX/AcOEt 9: 1.^{1}H NMR (600 MHz, $CDCl_3$) δ (ppm): 7.71 – 7.67 (m, 3H), 7.60 (d, J = 8.4 Hz, 1H), 7.55 (dd, J_1 = 1.5 Hz, J_2 = 8.4 Hz), 6.94 – 6.89 (m, 2H), 6.77 (d, J = 2.0 Hz, 2H), 6.70 (d, J = 2.2 Hz, 2H), 6.57 (t, J = 2.2 Hz, 1H), 6.49 (t, J = 2.0 Hz, 1H), 3.88 (s, 6H), 3.86 (s, 3H), 3.82 (s, 6H). ^{13}C NMR (150 MHz, $CDCl_3$) δ (ppm): 161.3 (2C), 161.0 (× 2C), 159.8, 153.1, 151.4, 144.0, 136.6. 134.8, 130.8, 128.5 (× 2C), 124.0, 123.1, 118.3, 116.1, 113.9 (× 2C), 111.0, 107.7 (× 2C), 105.9 (× 2C), 100.0, 98.8, 55.4 (× 4C), 55.3.

5,5'-(2-(4-Hydroxyphenyl)benzofuran-3,5-diyl)bis(benzene-1,3-diol) (10). To a solution of compound **9** (60 mg, 0.121 mmol, 1 eq) in dry DCM (1.2 mL) at -78 °C, 1M BBr_3 in DCM (0.67 mL, 0.67 mmol, 5 eq) was added dropwise and the resulting mixture was allowed to warm to room temperature and stirred overnight. The mixture was quenched with aq 5% $NaHCO_3$ at 0°C (pH 7).

The aqueous layer was extracted with EtOAc (3 × 10 mL). The combined organic layers were dried over anhydrous Na_2SO_4, filtered, and evaporated. The crude was purified on silica gel by column chromatography using as eluent DCM/MeOH 9:1. The product was obtained as a yellowish solid (96%). M.p.: 161 – 163 °C. R*f*: 0.25 DCM/MeOH 9:1. ^{1}H NMR (600 MHz, CD_3OD) δ (ppm): 7.66 – 7.48 (m, 5H), 6.84 – 6.76 (m, 2H), 6.58 (d, *J* = 2.0 Hz, 2H), 6.47 (d, *J* = 2.1 Hz, 2H), 6.35 (t, *J* = 2.0 Hz, 1H), 6.26 (t, *J* = 2.1 Hz, 1H). ^{13}C NMR (150 MHz, CD_3OD) δ (ppm): 160.2 (× 2C), 159.8 (× 2C), 159.2, 154.7, 152.9, 145.0, 137.8, 136.0, 131.9, 129.7 (× 2C), 124.5, 123.2, 118.7, 116.9, 116.3 (× 2C), 111.7, 109.1 (× 2C), 106.8 (× 2C), 102.9, 102.2. Anal. calcd. for $C_{26}H_{18}O_6$: C, 73.23; H, 4.25. Found: C, 73.11; H, 4.26.

Methyl 3-(3,5-dimethoxyphenyl)-2-(4-methoxyphenyl)benzofuran-5-carboxylate (12). Methyl 4-hydroxy-3-iodobenzoate **11** (100 mg, 0.360 mmol, 1 eq) and $PdCl_2(PPh_3)_2$ (7.57 mg, 0.011 mmol, 0.03 eq) were dissolved in dry THF (0.35 mL) in a microwave vial under N_2 atmosphere. Dry TEA (1.1 mL) and CuI (1.4 mg, 0.007 mmol, 0.02 eq) were added and the reaction mixture was stirred for 10 min. Then, 4-ethynilanisole **6** (56 µL, 0.432 mmol, 1.2 eq) was added and the mixture was stirred in a microwave reactor for 30 min at room temperature. After that, 1-iodo-3,5-dimethoxybenzene **7** (95 mg, 0.360 mmol, 1 eq) and dry acetonitrile (1.43 mL) were added and the mixture was stirred at 100 °C for 25 min, under microwave irradiation. The reaction mixture was allowed to cool to room temperature and the solvent was evaporated. The crude was purified on silica gel using as eluent CHX/DCM (from 1:1 to 3:7) to give the desired compound as a yellow solid (66% yield). M.p.: 175 – 177 °C. R*f*: 0.26 (CHX/DCM 1:1). ^{1}H NMR (300 MHz, $CDCl_3$) δ (ppm): 8.19 (dd, J_1= 0.6 Hz, J_2 = 1.8 Hz, 1H), 8.03 (dd, J_1= 1.8 Hz, J_2 = 8.6 Hz, 1H), 7.67 -7.60 (m, 2H), 7.54 (dd, J_1= 0.6 Hz, J_2 = 8.6 Hz, 1H), 6.91-6.83 (m, 2H), 6.63 (d, *J* = 2.3 Hz, 2H), 6.54 (t, *J* = 2.3 Hz, 1H), 3.91 (s, 3H), 3.83 (s, 3H), 3.79 (6H, s).^{13}C NMR (150 MHz, $CDCl_3$) δ (ppm): 167.3, 161.3 (× 2C), 160.0, 156.3, 152.0, 134.2, 130.5, 128.5 (× 2C), 126.1, 125.3, 122.6, 122.2, 116.2, 114.0 (× 2C), 110.8, 107.7 (× 2C), 100.1, 55.4 (× 2C), 55.3, 52.0.

3-(3,5-Dimethoxyphenyl)-2-(4-methoxyphenyl)benzofuran-5-carboxylic acid (13). Compound **12** (90 mg, 0.215 mmol, 1 eq) was dissolved in THF (2.8 mL). Then a solution of $LiOH{\cdot}H_2O$ (45 mg, 1.075 mmol, 5 eq) in water (2.8 mL) was added and the resulting suspension was stirred for 24h. When the reaction was completed the suspension became a clear solution. The organic solvent was evaporated and the aqueous phase was quenched at 0 °C with aq 1M HCl (pH 2-3). The aqueous phase was extracted with EtOAc three times. The combined organic phases were washed with brine, dried over anhydrous Na_2SO_4, filtered and evaporated. The product was obtained as a yellowish solid in quantitative yield. M.p.: 262 – 264°C. R*f*: 0.33 (CHX/AcOEt 3:2). ^{1}H NMR (600 MHz, DMSO-

d$_6$) δ (ppm): 12.91 (brs, 1H), 7.98 (d, *J* = 1.6 Hz, 1H), 7.94 (dd, J_1 = 1.6 Hz, J_2 = 8.6 Hz, 1H), 7.60 – 7.56 (m, 2H), 7.03 – 6.98 (m, 2H), 6.62 (t, *J* = 2.0 Hz, 1H), 6.60 (d, *J* = 2.0 Hz, 2H), 3.77 (s, 3H), 3.74 (s, 6H). ^{13}C NMR (150 MHz, DMSO-*d*$_6$) δ (ppm): 167.3, 161.1 (× 2C), 160.0, 155.4, 151.5, 133.4, 129.8, 128.3 (× 2C), 126.3, 126.1, 121.6, 121.2, 115.6, 114.3 (× 2C), 111.2, 107.4 (× 2C), 99.8, 55.3 (× 3C).

***N*, 3-bis(3,5-dimethoxyphenyl)-2-(4-methoxyphenyl)benzofuran-5-carboxamide (14)**. To a solution of compound **13** (48 mg, 0.119 mmol, 1 eq) in dry DMF (1 mL) at 0 °C, under N_2 atmosphere, EDC·HCl (34.4 mg, 0.179 mmol, 1.5 eq) and HOBt (24 mg, 0.179 mmol, 1.5 eq) were added. The ice-bath was removed and the mixture was stirred at room temperature for 1h. Then, the reaction was cooled again to 0 °C, and DIPEA (41 µL, 0.239 mmol, 2 eq) and 3,5-dimethoxyaniline (22 mg, 0.143 mmol, 1.2 eq) were added and the mixture was warmed to room temperature and stirred overnight. The reaction was quenched with aq 1M HCl (10 mL) and extracted with EtOAc (3 × 10 mL). The combined organic phases were washed wit aq saturated $NaHCO_3$ solution (20 mL), brine (2 × 20 mL), dried over anhydrous Na_2SO_4, filtered, and evaporated. The crude was purified on silica gel by column chromatography using as eluent CHX: AcOEt 7:3. The product was obtained as brownish solid (70%). M.p.: 166 – 167 °C. R*f*: 0.36 (CHX/AcOEt 7:3). ^{1}H NMR (600 MHz, $CDCl_3$) δ (ppm): 7.94 (d, *J* = 1.8 Hz, 1H), 7.83 (dd, J_1 = 8.6 Hz, J_2 = 2.0 Hz, 1H), 7.81 (brs, 1H), 7.69 – 7.62 (m, 2H), 7.59 (d, *J* = 8.6 Hz, 1H), 6.91 (d, *J* = 2.2 Hz, 2H), 6.90 – 6.82 (m, 2H), 6.63 (d, *J* = 2.3 Hz, 2H), 6.54 (t, *J* = 2.3 Hz, 1H), 6.27 (t, *J* = 2.2 Hz, 1H), 3.83 (s, 3H), 3.79 (s, 6H). ^{13}C NMR (150 MHz, $CDCl_3$) δ (ppm): 166.1, 161.4 (× 2C), 160.1 (× 2C), 160.1, 155.5, 152.3, 139.9, 134.2, 130.7, 130.2, 128.5 (× 2C), 123.7, 122.5, 118.9, 116.0, 114.0 (× 2C), 111.2, 107.7 (× 2C), 100.0, 98.3 (× 2C), 97.0, 55.4 (× 4C), 55.3.

***N*, 3-bis(3,5-dihydroxyphenyl)-2-(4-hydroxyphenyl)benzofuran-5-carboxamide (15)**. To a solution of compound **14** (35 mg, 0.0645 mmol, 1 eq) in dry DCM (1.2 mL) at -78 °C, 1M BBr_3 in DCM (0.43 mL, 0.43 mmol, 6.6 eq) was added dropwise and the resulting mixture was allowed to warm to room temperature and stirred overnight. The mixture was quenched with aq 5% $NaHCO_3$ at 0 °C (pH 7). The aqueous layer was extracted with EtOAc (3 × 10 mL). The combined organic layers were dried over anhydrous Na_2SO_4, filtered, and evaporated. The crude was purified on silica gel by column chromatography using as eluent DCM/MeOH 85:15. The product was obtained as brownish sticky solid (73% yield). M.p.: 199 – 201 °C. R*f*: 0.36 DCM/MeOH 85:15. ^{1}H NMR (600 MHz, CD_3OD) δ (ppm): 8.06 (d, *J* = 1.6 Hz, 1H), 7.88 (dd, J_1 = 8.8 Hz, J_2 = 1.6 Hz), 7.63 (d, *J* = 8.8 Hz, 1H), 7.61 – 7.55 (m, 2H), 6.85 – 6.78 (m, 2H), 6.74 (d, *J* = 1.8 Hz, 2H), 6.46 (d, *J* = 2.0 Hz, 2H), 6.36 (t, *J* = 2.0 Hz, 1H), 6.10 (t, *J* = 1.8 Hz, 1H). ^{13}C NMR (150 MHz, CD_3OD) δ (ppm): 169.2, 160.3 (×

2C), 159.6 (× 2C), 159.5, 156.9, 153.8, 141.4, 135.5, 131.7, 131.5, 129.8 (× 2C), 125.1, 122.7, 120.8, 117.0, 116.4 (× 2C), 111.7, 109.2 (× 2C), 103.2, 101.2 (× 2C), 100.1. Anal. calcd. for $C_{27}H_{19}NO_7$: C, 69.08; H, 4.08; N, 2.98. Found: C, 69.18; H, 4.07; N, 2.99.

(3-(3,5-Dimethoxyphenyl)-2-(4-methoxyphenyl)benzofuran-5-yl)methanol (16). To a solution of compound **12** (220 mg, 0.526 mmol, 1 eq) in dry THF (5.3 mL) at 0 °C, under N_2 atmosphere, $LiAlH_4$ 1M in THF (1.58 mL, 158 mmol, 3 eq) was added dropwise and the yellow solution was stirred for 20 min at 0 °C. Then, the reaction mixture was quenched with aq 1M HCl at 0 °C and the aqueous layer was extracted with EtOAc three times. The combined organic layers were dried over anhydrous Na_2SO_4, filtered and evaporated. The crude was purified on silica gel by column chromatography using as eluent CHX/AcOEt 6:4 to afford the product as a yellow foamy solid in quantitative yield. Analytical data agreed with data reported in literature [40]. R_f: 0.44 (CHX/AcOEt 6:4). ^{1}H NMR (600 MHz, $CDCl_3$) δ (ppm): 7.64 (d, J = 8.9 Hz, 2H), 7.51 (d, J = 8.3 Hz, 1H), 7.49 (d, J = 1.6 Hz, 1H), 7.32 (dd, J_1 = 8.3, J_2 = 1.8 Hz, 1H), 6.87 (d, J = 8.9 Hz, 2H), 6.63 (d, J = 2.3 Hz, 2H), 6.52 (t, J = 2.3 Hz, 1H), 4.75 (s, 2H), 3.82 (s, 3H), 3.78 (s, 6H). ^{13}C NMR (150 MHz, $CDCl_3$) δ (ppm): 161.4 (× 2C), 160.0, 153.5, 151.5, 135.9, 135.0, 130.7 (× 2C), 128.6, 124.0, 123.2, 118.7, 116.1, 114.1 (× 2C), 111.2, 107.9 (× 2C), 100.0, 65.9, 55.6 (× 3C).

Diethyl ((3-(3,5-dimethoxyphenyl)-2-(4-methoxyphenyl)benzofuran-5-yl)methyl)phosphonate (17). To a suspension of compound **16** (200 mg, 0.512 mmol, 1 eq) in dry Et_2O (2.6 mL) at room temperature, under N_2 atmosphere, catalytic pyridine (2 µL, 0.026 mmol, 0.05 eq) was added, followed by the dropwise addition of PBr_3 (40 µL, 0.5122 mmol, 1 eq), and the mixture was stirred at reflux (40 °C) for 90 min. The reaction was cooled to room temperature and quenched with ice and water. The aqueous phase was extracted with EtOAc three times. The combined organic layers were washed with brine twice, dried over anhydrous Na_2SO_4, filtered and evaporated, to afford 5-(bromomethyl)-3-(3,5-dimethoxyphenyl)-2-(4-methoxyphenyl)benzofuran as a yellow oil. The product was used immediately for the next step without any further purification. R_f: 0.41 CHX: AcOEt 9:1.

The brominated intermediate (182 mg, 0.401 mmol, 1 eq) was dissolved with triethylphosphite (200 µL, 1.166 mmol, 2.9 eq) and heated at 130 °C overnight. Triethylphosphite in excess was evaporated and the crude was purified by flash column chromatography, using as eluent CHX/AcOEt 3:7. The product was obtained as a yellow oil in 80% yield over two steps. R_f: 0.19 CHX/AcOEt 3: 7. ^{1}H NMR (300 MHz, $CDCl_3$) δ (ppm): 7.65 – 7.60 (m, 2H), 7.56 (s, 1H), 7.45 (d, J = 8.3 Hz, 1H), 7.38 (d, J = 2.1 Hz, 1H), 7.25 (dd, J_1 = 8.3 Hz, J_2 = 2.1 Hz), 6.88 – 6.83 (m, 2H), 6.61 (d, J = 2.2 Hz, 2H), 6.50 (t, J = 2.2 Hz, 1H), 4.05 – 3.95 (m, 4H), 3.82 (s, 3H), 3.78 (s, 6H), 3.21 (d, J = 21.0 Hz, 2H), 1.25 –

1.20 (m, 6H). ^{13}C NMR (150 MHz, $CDCl_3$) δ (ppm): 161.2 (× 2C), 159.8, 152.9, 151.2, 134.8, 130.6, 128.5 (× 2C), 126.1, 126.0, 123.1, 120.8, 115.8, 113.9 (× 2C), 110.8, 107.7 (× 2C), 99.8, 62.1, 62.0, 55.4 (× 2C), 55.3, 33.6 (d, *J (C, P)* = 138 Hz), 16.4, 16.3.

(E)-3-(3,5-Dimethoxyphenyl)-2-(4-methoxyphenyl)-5-(4-methoxystyryl)benzofuran (18). In a microwave vial, a suspension of compound **17** (70 mg, 0.135 mmol, 1.5 eq) with *p*-anisaldehyde (11 µL, 0.091 mmol, 1 eq) and 60% NaH (11 mg, 0.274 mmol, 3 eq) in dry THF (1.6 mL) was stirred at 120 °C for 30 min under microwave irradiation. The reaction mixture was cooled to room temperature, and quenched with aq saturated NH_4Cl. The aqueous phase was extracted with EtOAc three times. The combined organic layers were washed with water, brine, dried over anhydrous Na_2SO_4, filtered, and evaporated. The crude was purified on silica gel by column chromatography, using as eluent CHX/AcOEt (from 9:1 to 85:15) to yield the product as a golden foamy solid (86%). R_f: 0.32 (CHX/AcOEt 85:15). ^1H NMR (600 MHz, $CDCl_3$) δ (ppm): 7.65 – 7.61 (m, 2H), 7.56 (s, 1H), 7.51 – 7.46 (m, 2H), 7.45 – 7.42 (m, 2H), 7.06 (d, *J* = 16.1 Hz, 1H), 7.01 (d, *J* = 16.1 Hz, 1H), 6.91 – 6.88 (m, 2H), 6.88 – 6.84 (m, 2H), 6.66 (d, *J* = 1.6 Hz, 2H), 6.54 (t, *J* = 1.6 Hz, 1H), 3.83 (s, 6H), 3.80 (s, 6H). ^{13}C NMR (150 MHz, $CDCl_3$) δ (ppm): 161.2 (x2C), 159.7, 159.1, 153.3, 151.3, 134.9, 132.9, 130.8, 130.4, 128.4 (× 2C), 127.5 (× 2C), 127.1, 126.9, 123.1, 122.9, 117.3, 116.0, 114.1 (× 2C), 113.9 (× 2C), 111.0, 107.7 (× 2C), 99.9, 55.4 (× 2C), 55.3 (× 2C).

2-Iodo-4-methylphenol (22). To a solution of *para*-cresol **21** (500 mg, 4.6236 mmol, 1 eq) in dry ACN (4.62 mL) at room temperature, under N_2 atmosphere, *para*-toluensulfonic acid monohydrate (880 mg, 4.6236 mmol, 1 eq) was added and the suspension was stirred for 10 min to give a clear solution. Then, *N*-iodosuccinimide (1.037 g, 4.6236 mmol, 1 eq) was added and the reaction mixture was stirred overnight at room temperature. The reaction was quenched with aq 20% $Na_2S_2O_5$ (100 mL) and extracted with EtOAc (3 × 70 mL). The combined organic layers were washed with brine, dried over anhydrous Na_2SO_4, filtered and evaporated. The crude was purified on silica gel using as eluent CHX/DCM (from 1:1 to 4:6) to afford the desired product as transparent oil in 97% yield. Analytical data were consistent with data reported in literature [36]. R_f: 0.35 (CHX/DCM 1:1).^1H NMR (600 MHz, $CDCl_3$) δ (ppm): 7.48 (d, *J* = 1.3 Hz, 1H), 7.04 (dd, J_1 = 8.2 Hz, J_2 = 1.5 Hz, 1H), 6.88 (d, *J* = 8.2 Hz, 1H), 5.21 (s, 1H), 2.25 (s, 3H). ^{13}C NMR (150 MHz, $CDCl_3$) δ (ppm): 152.6, 138.4, 132.1, 131.0, 114.8, 85.5, 20.1.

3-(3,5-Dimethoxyphenyl)-2-(4-methoxyphenyl)-5-methylbenzofuran (23). Compound **22** (100 mg, 0.427 mmol, 1 eq) and $PdCl_2(PPh_3)_2$ (9 mg, 0.013 mmol, 0.03 eq) were dissolved in dry THF (0.43 mL) in a microwave vial, under N_2 atmosphere. Dry TEA (1.2 mL) and CuI (1.6 mg, 0.008 mmol, 0.02 eq) were added and the reaction mixture was stirred for 10 min. Then, 4-ethynilanisole **6**

(67 µL, 0.513 mmol, 1.2 eq) was added and the mixture was stirred in a microwave reactor for 30 min at room temperature. After that, 1-iodo-3,5-dimethoxybenzene **7** (95 mg, 0.360 mmol, 1 eq) and dry acetonitrile (1.43 mL) were added and the mixture was stirred at 100 °C for 25 min, under microwave irradiation. The reaction mixture was allowed to cool to room temperature and the solvent was evaporated. The crude was purified on silica gel using as eluent CHX/DCM (from 1:1 to 4:6) to give the desired compound as a sticky yellow solid (48% yield). R_f: 0.29 (CHX: DCM 6:4). ^{1}H NMR (300 MHz, $CDCl_3$) δ (ppm): 7.65 – 7.61 (m, 2H), 7.40 (d, J = 8.2 Hz, 1H), 7.28 (d, J = 1.5 Hz, 1H), 7.11 (dd, J_1 = 8.2 Hz, J_2 = 1.5 Hz, 1H), 6.88 – 6.84 (m, 2H), 6.64 (d, J = 2.2 Hz, 2H), 6.51 (t, J = 2.2 Hz, 1H), 3.82 (s, 3H), 3.79 (s, 6H), 2.42 (s, 3H). ^{13}C NMR (150 MHz, $CDCl_3$) δ (ppm): 161.2 (× 2C), 160.5, 152.1, 150.8, 135.2, 132.3, 130.4, 128.4 (× 2C), 125.5, 123.3, 119.6, 115.7, 113.8 (× 2C), 110.4, 107.7, 99.8, 55.4 (× 2C), 55.3 (× 2C), 21.4.

5-(2-(4-Hydroxyphenyl)-5-methylbenzofuran-3-yl)benzene-1,3-diol (24). To the solution of compound **23** (75 mg, 0.200 mmol, 1 eq) in dry DCM (2 mL) at – 78 °C, 1M BBr_3 in DCM (0.66 mL, 0.660 mmol, 3.3 eq) was added dropwise and the resulting mixture was allowed to warm to room temperature and stirred overnight. The mixture was quenched with aq 5% $NaHCO_3$ at 0 °C (pH 7). The aqueous layer was extracted with EtOAc (3 × 10 mL). The combined organic layers were dried over anhydrous Na_2SO_4, filtered, and evaporated. The crude was purified on silica gel by column chromatography using as eluent DCM/ MeOH 95:5. The product was obtained as yellow solid in 90% yield. M.p.: 58 – 60°C. R_f: 0.34 DCM/MeOH 95:5. ^{1}H NMR (600 MHz, CD_3OD) δ (ppm): 7.54 – 7.51 (m, 2H), 7.37 (d, J = 8.4 Hz, 1H), 7.26 (d, J = 1.5 Hz, 1H), 7.11 (dd, J_1 = 8.4 Hz, J_2 = 1.5 Hz, 1H), 6.79 – 6.75 (m, 2H), 6.41 (d, J = 2.2 Hz, 2H), 6.33 (t, J = 2.2 Hz, 1H), 2.42 (s, 3H). ^{13}C NMR (150 MHz, CD_3OD) δ (ppm): 160.1 (× 2C), 159.0, 153.5, 152.2, 136.3, 133.4, 131.6, 129.6 (× 2C), 126.3, 123.5, 120.4, 116.6, 116.2 (× 2C), 111.1, 109.1 (× 2C), 102.8, 21.4.

((5-(2-(4-((*tert*-Butyldimethylsilyl)oxy)phenyl)-5-methylbenzofuran-3-yl)-1,3-phenylene)bis(oxy))bis(*tert*-butyldimethylsilane) (25). Imidazole (277 mg, 4.062 mmol, 4.5 eq) and TBDMSCl (531 mg, 3.520 mmol, 3.9 eq) were added to a suspension of compound **24** (300 mg, 0.903 mmol, 1 eq) in 1,2-dichloroethane (9 mL), and the resulting mixture was stirred at 60 °C overnight, under N_2 atmosphere. The mixture was allowed to cool to room temperature and quenched with brine. The aqueous phase was extracted with EtOAc three times. The combined organic layers were dried over anhydrous Na_2SO_4, filtered, and evaporated. The crude was purified on silica gel by column chromatography, using as eluent CHX/DCM (from 100% to 95%), to afford the desired product as foamy white solid in 86% yield. R_f: 0.51 CHX: DCM 95:5. ^{1}H NMR (600 MHz, $CDCl_3$) δ (ppm): 7.57 – 7.53 (m, 2H), 7.39 (d, J = 8.2 Hz, 1H), 7.23 (d, J = 1.3 Hz, 1H), 7.10 (dd, J_1 = 8.2

Hz, J_2 = 1.3 Hz), 6.79 – 6.75 (m, 2H), 6.58 (d, J = 2.3 Hz, 2H), 6.40 (t, J = 2.3 Hz, 1H), 2.42 (s, 3H), 0.98 (s, 27H), 0.20 (s, 6H), 0.18 (s, 12H). ^{13}C NMR (150 MHz, $CDCl_3$) δ (ppm): 157.1 (× 2C), 155.8, 152.1, 150.7, 134.8, 132.2, 130.5, 128.3 (× 2C), 125.3, 124.0, 120.1 (× 2C), 119.6, 115.6, 114.9 (× 2C), 111.7, 110.4, 25.6 (× 9C), 21.3, 18.2 (× 3C), -4.4 (× 6C).

Diethyl ((3-(3,5-bis((tert-butyldimethylsilyl)oxy)phenyl)-2-(4-((*tert*-butyldimethylsilyl)oxy)phenyl)benzofuran-5-yl)methyl)phosphonate (26). To a solution of compound **25** (500 mg, 0.74 mmol, 1 eq) in CCl_4 (7.4 mL), *N*-bromosuccinimide (145 mg, 0.8146 mmol, 1.1 eq) and AIBN (12 mg, 0.074 mmol, 0.1 eq) were added and the mixture was refluxed for 8h, under N_2 atmosphere. The reaction mixture was cooled to room temperature, filtered and the filtrate was evaporated. The crude was purified on silica gel using as eluent CHX/DCM (from 95% to 90%) to obtain ((5-(5-(bromomethyl)-2-(4-((*tert*-butyldimethylsilyl)oxy)phenyl)benzofuran-3-yl)-1,3-phenylene)bis(oxy))bis(*tert*-butyldimethylsilane) as a transparent oil (37%). R_f: 0.2 CHX/DCM 98:2. ^{1}H NMR (600 MHz, $CDCl_3$) δ (ppm): 7.58 – 7.52 (m, 2H), 7.48 – 7.44 (m, 2H), 7.33 (dd, J_1 = 8.4 Hz, J_2 = 1.3 Hz), 6.79 – 6.75 (m, 2H), 6.57 (d, J = 1.9 Hz, 2H), 6.42 (t, J = 1.9 Hz, 1H), 4.60 (s, 2H), 0.98 (s, 27H), 0.20 (s, 6H), 0.19 (s, 12H). ^{13}C NMR (150 MHz, $CDCl_3$) δ (ppm): 157.2 (× 2C), 156.1, 153.5, 151.6, 134.2, 132.6, 130.9, 128.4 (× 2C), 125.5, 123.5, 120.4, 120.1 (× 2C), 115.7, 114.8 (× 2C), 111.9, 111.2, 34.4, 25.6 (× 9C), 18.2 (× 3C), -4.3 (× 4C), -4.4 (× 2C).

The above compound (150 mg, 0.199 mmol, 1 eq) in triethylphosphite (0.2 mL, 1.167 mmol, 5.9 eq) was heated at 130 °C overnight in a sealed vial. Triethylphosphite was evaporated and the crude was purified on silica gel by column chromatography using as eluent CHX/AcOEt (6:4) to afford the product as a transparent oil in 84% yield. R_f: 0.33 CHX/AcOEt 6:4. ^{1}H NMR (600 MHz, $CDCl_3$) δ (ppm): 7.56 – 7.52 (m, 2H), 7.44 (d, J = 8.5 Hz, 1H), 7.30 – 7.26 (m, 2H), 6.80 – 6.75 (m, 2H), 6.56 (d, J = 2.2 Hz, 2H), 6.40 (t, J = 2.2 Hz, 1H), 4.04 – 3.94 (m, 4H), 3.21 (d, J = 21.0 Hz, 2H), 1.22 (t, J = 7.0 Hz, 6H), 1.00 – 0.95 (m, 27H), 0.20 (s, 6H), 0.17 (s, 12H). ^{13}C NMR (600 MHz, $CDCl_3$) δ (ppm): 157.1 (× 2C), 156.0, 152.8, 151.1, 134.5, 130.7, 128.3 (× 2C), 125.9, 123.7, (× 2C), 120.8, 120.1 (× 2C), 115.7, 114.9 (× 2C), 111.7, 110.9, 62.1 (× 2C), 31.9, 25.6 (× 9C), 18.2 (× 3C), 16.4 (× 2C), -4.6 (× 6C).

(*E*)-4-(2-(3-(3,5-Dihydroxyphenyl)-2-(4-hydroxyphenyl)benzofuran-5-yl)vinyl)benzene-1,2-diol (27). To a solution of compound **26** (80 mg, 0.099 mmol, 1 eq) in dry THF (0.86 mL) at 0 °C, under nitrogen atmosphere, 60% NaH (8 eq, 0.8 mmol, 32 mg) was added and the mixture was stirred at 0 °C for 45 min. Then, a solution of 3,4-bis((*tert*-butyldimethylsilyl)oxy)benzaldehyde (188 mg, 0.514 mmol, 5.2 eq) was added dropwise and the solution was allowed to warm to room temperature

and stirred overnight. The reaction mixture was quenched with aq 5% $NaHCO_3$. The aqueous layer was extracted with EtOAc five times. The combined organic layers were dried over anhydrous Na_2SO_4, filtered, and evaporated. The crude was purified on silica gel using as eluent CHX/DCM 95:5 to afford (*E*)-((4-(2-(3-(3,5-bis((*tert*-butyldimethylsilyl)oxy)phenyl)-2-(4-((*tert*-butyldimethylsilyl)oxy)phenyl)benzofuran-5-yl)vinyl)-1,2-phenylene)bis(oxy))bis(*tert*-butyldimethylsilane) as transparent sticky solid in 52% yield. R_f: 0.65 CHX/DCM 95:5. ^{1}H NMR (600 MHz, $CDCl_3$) δ (ppm): 7.59 – 7.55 (m, 2H), 7.54 (s, 1H), 7.46 (d, J = 8.5 Hz, 1H), 7.44 (d, J = 8.5 Hz, 1H), 7.00 – 6.94 (m, 4H), 6.93 (d, J = 16.5 Hz, 1H), 6.81 (d, J = 7.9 Hz, 1H), 6.79 – 6.76 (m, 2H), 6.61 (d, J = 1.8 Hz, 2H), 6.43 (t, J = 1.8 Hz, 2H), 1.05 – 0.95 (m, 45H), 0.23 (s, 6H), 0.21 (s, 6H), 0.20 (s, 18H). ^{13}C NMR (600 MHz, $CDCl_3$) δ (ppm): 157.1 (× 2C), 156.0, 153.3, 151.2, 146.9, 146.6, 134.6, 132.8, 131.3, 130.9, 128.4 (× 2C), 127.2, 127.0, 123.7, 123.1, 121.1, 120.1 (x2C), 119.7, 118.9, 117.0, 115.9, 114.9 (× 2C), 111.9, 110.9, 26.0 (× 3C), 25.9 (× 3C), 25.7 (× 6C), 25.6 (× 3C), 18.5 (× 2C), 18.2 (× 3C), -4.0 (× 2C), - 4.1 (× 2C), -4.3 (× 4C), - 4.4 (× 2C).

To a solution of the above compound (26 mg, 0.026 mmol, 1 eq) in dry THF (0.5 mL) TBAF 1M in THF (0.169 mL, 0.169 mmol, 6.5 eq) was added at 0 °C and the reaction mixture was stirred at room temperature for 90 min. The mixture was quenched with water and the aqueous layer was extracted with EtOAc three times. The combined organic layers were washed with aq 0.1 M HCl three times, dried over anhydrous Na_2SO_4, filtered, and evaporated. The crude was purified on silica gel by column chromatography to afford the desired product in 60 % yield, as a transparent sticky solid. R_f: 0.43 DCM/MeOH 9:1. ^{1}H NMR (600 MHz, CD_3OD) δ (ppm): 7.56 – 7.51 (m, 3H), 7.48 (dd, J_1 = 8.6 Hz, J_2 = 1.5 Hz, 1H), 7.45 (d, J = 8.6 Hz, 1H), 7.01 (d, J = 16.1 Hz, 1H), 7.00 (d, J = 1.5 Hz, 1H), 6.95 (d, J = 16.1 Hz, 1H), 6.86 (dd, J_1 = 8.0 Hz, J_2 = 1.5 Hz), 6.79 – 6.75 (m, 2H), 6.73 (d, J = 8.0 Hz, 1H), 6.43 (d, J = 2.1 Hz, 1H), 6.34 (t, J = 2.1 Hz, 1H). ^{13}C NMR (150 MHz, CD_3OD) δ (ppm): 160.2 (× 2C), 159.2, 154.6, 152.7, 146.5, 146.3, 136.1, 134.6, 132.0, 131.4, 129.6 (× 2C), 128.8, 127.1, 123.7, 123.3, 120.1, 118.1, 116.9, 116.4, 116.3 (× 2C), 113.7, 111.8, 109.2 (× 2C), 103.0. Anal. calcd. for $C_{28}H_{20}O_6$: C, 74.33; H, 4.46. Found: C, 74.45; H, 4.46.

((5-(5-Bromo-2-(4-((*tert*-butyldimethylsilyl)oxy)phenyl)benzofuran-3-yl)-1,3-phenylene)bis(oxy))bis(*tert*-butyldimethylsilane) (28). To a solution of compound **8** (700 mg, 1.593 mmol, 1 eq) in dry DCM (15.9 mL), under nitrogen atmosphere, at -78 °C, BBr_3 1M in DCM (5.3 mL, 5.3 mmol, 3.3 eq) was added dropwise, and the resulting mixture was slowly allowed to warm to room temperature and stirred overnight. The reaction mixture was quenched at 0 °C with aq 5% $NaHCO_3$ (pH 7). The aqueous layer was extracted with EtOAc three times. The combined organic layers were dried over anhydrous Na_2SO_4, filtered, and evaporated. The crude was purified on silica

gel by column chromatography, using DCM/MeOH (95:5) as eluent to afford 5-(5-bromo-2-(4-hydroxyphenyl)benzofuran-3-yl)benzene-1,3-diol as a brownish amorphous solid in 87% yield. R_f: 0.27 (DCM/MeOH 95:5).^{1}H NMR (600 MHz, CD_3OD) δ (ppm): 7.55 – 7.52 (3H, m), 7.44 (d, J = 8.6 Hz, 1H), 7.39 (dd, J_1 = 1.9 Hz, J_2 = 8.6 Hz), 6.80 – 6.76 (2H, m), 6.39 (d, J = 2.2 Hz, 2H), 6.34 (t, J = 2.2 Hz, 1H). ^{13}C NMR (600 MHz, CD_3OD) δ (ppm): 160.3 (× 2C), 159.6, 153.8, 153.7, 135.3, 133.7, 129.8 (× 2C), 127.9, 123.1, 122.6, 116.9, 116.4 (× 2C), 116.2, 113.4, 108.9 (× 2C), 103.2.

Following a procedure by Romero *et al* [8], imidazole (131 mg, 1.926 mmol, 4.5 eq) and TBDMSCl (252 mg, 1.669 mmol, 3.9 eq) were added to a suspension of the above compound (170 mg, 0.428 mmol, 1 eq) in 1,2-dichloroethane (4.3 mL), and the resulting mixture was stirred at 60 °C for 8h, under N_2 atmosphere. The mixture was allowed to cool to room temperature and quenched with brine. The aqueous phase was extracted three times with EtOAc. The combined organic layers were dried over anhydrous Na_2SO_4, filtered, and evaporated. The crude was purified on silica gel by column chromatography, using as eluent CHX/DCM 95:5 to afford the desired product as foamy white solid in 81% yield. R_f: 0.36 CHX/DCM 95:5.^{1}H NMR (600 MHz, $CDCl_3$) δ (ppm): 7.58 – 7.53 (m, 3H), 7.39 – 7.37 (m, 2H), 6.81 – 6.76 (m, 2H), 6.54 (d, J = 2.4 Hz, 2H), 6.41 (t, J = 2.4 Hz, 1H), 0.98 (s, 27H), 0.19 (s, 18H). ^{13}C NMR (150 MHz, $CDCl_3$) δ (ppm): 158.6 (× 2C), 157.7, 153.8, 153.2, 135.3, 133.9, 129.8 (× 2C), 128.3, 124.6, 123.8, 121.6 (× 2C), 117.3, 116.7, 116.1 (× 2C), 113.7, 113.4, 27.0 (× 9C), 19.6 (× 3C), -2.9 (× 6C).

((5-(2,2-Dibromovinyl)-1,3-phenylene)bis(oxy))bis(*tert*-butyldimethylsilane) (30). To a solution of 3,5-dihydroxybenzaldehyde **29** (300 mg, 2.172 mmol, 1 eq) in dry DMF (8 mL) at 0 °C, under N_2 atmosphere, imidazole (739 mg, 10.86 mmol, 5 eq) and *tert*-butyldimethylsilylchloride (786 mg, 5.212 mmol, 2.4 eq) were added, and the resulting mixture was stirred at 0 °C for 15 min and then at room temperature overnight. The reaction mixture was diluted with EtOAC (30 mL) and washed with a mixture of aq 0.1M HCl/brine 1:1 (5 x 20 mL). The organic phase was dried over anhydrous Na_2SO_4, filtered, and evaporated. The crude was purified on silica gel by column chromatography using as eluent CHX/AcOEt (from 100% to 98%). The product 3,5-bis(tert-butyldimethylsilyloxy)benzaldehyde was obtained as a transparent oil in 74% yield. The analytical data were in agreement with previous data reported in literature [41]. R_f: 0.63 CHX/AcOEt 98:2. ^{1}H NMR (300 MHz, $CDCl_3$) δ (ppm): 9.86 (s, 1 H), 6.95 (d, J= 2.1 Hz, 1 H), 6.58 (t, J = 2.1 Hz, 1 H), 0.98 (s, 18 H), 0.21 (s, 12 H). ^{13}C NMR (75 MHz, $CDCl_3$) δ (ppm): 191.6, 157.6 (× 2C), 138.8, 118.6, 114.6 (× 2C), 25.9 (× 2C), 18.5 (× 6C), -4.1 (× 4C).

To a solution of CBr_4 (1.067 g, 3.2183 mmol, 2 eq) in dry DCM (5.7 mL) at 0 °C, PPh_3 (1.688 g, 6.437 mmol, 4 eq) was added and the orange mixture was stirred for 10 min at 0 °C. Then, a solution of the above compound (590 mg, 1.609 mmol, 1 eq) in dry DCM (16 mL) was slowly added at 0 °C, and the resulting mixture was stirred for 10 min at the same temperature and 10 min at room temperature. The reaction was quenched with water (30 mL) and the aqueous phase was extracted with DCM (3 × 30 mL). The combined organic layers were dried over anhydrous Na_2SO_4, filtered, and evaporated. The crude was purified on silica gel by flash column chromatography, using as eluent CHX/DCM (from 95% to 90%). The product was obtained as a transparent oil in 90% yield. The analytical data agreed with previous data reported in literature [37]. R_f: 0.75 CHX: DCM 9:1.^{1}H NMR (300 MHz, $CDCl_3$) δ (ppm): 7.33 (s, 1 H), 6.63 (d, J = 2.2 Hz, 2 H), 6.31 (t, J = 2.2 Hz, 1 H), 0.95 (s, 18 H), 0.18 (s, 12 H). ^{13}C NMR (75 MHz, $CDCl_3$) δ (ppm): 156.47 (× 2C), 136.7, 136.6, 113.4 (× 2C), 112.7, 89.4, 25.7 (× 6 C), 18.2 (× 2C), −4.4 (× 4C).

((5-Ethynyl-1,3-phenylene)bis(oxy))bis(tert-butyldimethylsilane) (31). To a solution of compound **30** (340 mg, 0.651 mmol, 1 eq) in dry THF (7.5 mL) at -78 °C, under N_2 atmosphere, LDA 1M in THF (1.95 mL, 1.95 mmol, 3 eq) was added dropwise and the solution was stirred at -78 °C for 1h. The reaction was cautiously quenched with water and the aqueous phase was extracted with EtOAc three times. The combined organic layers were dried over anhydrous Na_2SO_4, filtered, and evaporated. The crude was purified on silica gel by column chromatography, using as eluent CHX/DCM (from 100% to 95%) to afford the desired product as transparent oil (91%). The analytical data were in agreement with previous data reported in literature [37]. R_f: 0.47 CHX/DCM 95:5. ^{1}H NMR (300 MHz, $CDCl_3$) δ (ppm): 6.59 (d, J = 2.2 Hz, 2 H), 6.34 (t, J = 2.2 Hz, 1 H), 2.99 (s, 1 H), 0.96 (s, 18 H), 0.18 (s, 12 H).^{13}C NMR (75 MHz, $CDCl_3$) δ (ppm): 156.4 (× 2C), 123.1, 117.2 (× 2C), 113.7, 83.5, 76.5, 25.6 (× 6C), 18.2 (× 2C), −4.4 (× 4C).

5-((3-(3,5-Dihydroxyphenyl)-2-(4-hydroxyphenyl)benzofuran-5-yl)ethynyl)benzene-1,3-diol (32). Compound **28**(240 mg, 0.324 mmol, 1 eq) and compound **31** (130 mg, 0.3564 mmol, 1.1 eq) were dissolved in previously degassed dry TEA (5 mL). $Pd(PPh_3)_4$ (11.2 mg, 0.0097 mmol, 0.03 eq) was added and the mixture was stirred for 10 min. Then, CuI (1.23 mg, 0.0065 mmol, 0.02 eq) was added and the mixture was refluxed for 8h, under N_2 atmosphere. The solvent was evaporated, and the resulting black residue was suspended in MeOH (6 mL). A solution of KF (280 mg, 4.86 mmol, 15 eq) in a mixture of MeOH/THF 1:1 (12 mL) was added dropwise. The reaction mixture was stirred overnight at room temperature. The solvents were evaporated and the residue was dissolved with some drops of MeOH and diluted with AcOEt. The organic phase was washed with a mixture of water/brine 1:1 (3 × 20 mL) and brine (3 × 20 mL). The organic layer was dried over anhydrous

Na_2SO_4, filtered, and evaporated. The crude was purified on silica gel by column chromatography, using as eluent DCM/MeOH 9:1. A fraction containing some impurities was further purified on silica gel by column chromatography using as eluent CHX/Acetone 1:1. The product was obtained as a yellow foamy solid in 38% yield over two steps. M.p.: 164 – 165°C. R_f: 0.23 DCM/MeOH 9:1 or 0.38 CHX/Acetone 1:1. ^1H NMR (300 MHz, CD_3OD) δ (ppm): 7.62 – 7.47 (m, 4H), 7.41 (dd, J_1 = 1.6 Hz, J_2 = 8.5 Hz, 1H), 6.77 (d, J = 8.8 Hz, 2H), 6.43 (d, J = 2.2 Hz, 2H), 6.41 (d, J = 2.2 Hz, 2H), 6.33 (t, J = 2.2 Hz, 1H), 6.26 (t, J = 2.3 Hz, 1H). ^{13}C NMR (150 MHz, CD_3OD) δ (ppm): 160.3 (× 2C), 159.7 (× 3C), 154.8, 153.5, 135.6, 133.0, 129.9 (× 2C), 128.8, 125.9, 124.0, 122.9, 119.5, 116.4 (× 2C), 112.1, 111.7, 110.9 (× 2), 109.1 (× 2C), 104.4, 103.2, 89.5, 89.2. Anal. calcd. for $C_{28}H_{18}O_6$: C, 74.66; H, 4.03. Found: C, 74.40; H, 4.02.

5-(5-(3,5-Dihydroxyphenethyl)-2-(4-hydroxyphenyl)benzofuran-3-yl)benzene-1,3-diol (33). To a solution of **1** (40 mg, 0.0884 mmol, 1 eq) in EtOH (2 mL), Pd/C 10% wt (4 mg) was added and the reaction mixture was stirred under H_2 atmosphere for 4 h at room temperature. The mixture was filtered on a celite pad, washing repeatedly the filter with methanol. The filtrate was evaporated to yield the product as an amorphous reddish solid in quantitative yield. R_f: 0.33 (DCM/MeOH 9:1). ^1H NMR (600 MHz, CD_3OD) δ (ppm): 7.50 – 7.46 (m, 2H), 7.34 (d, J = 8.4 Hz, 1H), 7.25 (d, J = 1.5 Hz, 1H), 7.06 (dd, J_1 = 1.5 Hz, J_2 = 8.4 Hz), 6.71 – 6.67 (m, 2H), 6.36 (d, J = 2.0 Hz, 1H), 6.28 (t, J = 2.0 Hz, 1H), 6.13 (d, J = 1.9 Hz, 2H), 6.08 (t, J = 2.0 Hz, 1H), 2.91 (t, J = 7.9 Hz, 2H), 2.75 (t, J = 7.9 Hz, 2H). ^{13}C NMR (150 MHz, CD_3OD) δ (ppm): 160.9, 160.3 (× 2C), 159.2 (× 2C), 153.3, 152.2, 145.0, 137.3, 135.9, 131.4, 129.2 (× 2C), 125.2, 121.8, 119.6, 116.6 (× 2C), 116.0, 110.6, 108.8 (× 2C), 107.7 (× 2C), 102.8, 101.1, 39.5, 38.6. Anal. calcd. for $C_{28}H_{22}O_6$: C, 74.00; H, 4.88. Found: C, 73.95; H, 4.89.

5-(1-(5-(3,5-Dihydroxyphenethyl)-2-hydroxyphenyl)-2-(4-hydroxyphenyl)ethyl)benzene-1,3-diol (35). To a solution of **34** (40 mg, 0.088 mmol, 1 eq) in EtOH (2 mL), Pd/C 10% wt (4 mg) was added and the reaction mixture was stirred under H_2 atmosphere for 4 h at room temperature. The mixture was filtered on a celite pad, washing repeatedly the filter with methanol. The filtrate was evaporated to yield the product as a reddish solid in quantitative yield. M.p.: 70 °C dec. R_f: 0.27 (DCM/MeOH 9:1). ^1H NMR (600 MHz, CD_3OD) δ (ppm): 7.01 (d, J = 2.2 Hz, 1H), 6.92-6.85 (m, 2H), 6.77 (dd, J_1= 8.1 Hz, J_2 = 2.2 Hz, 1H), 6.62-6.53 (m, 3H), 6.22 (d, J = 2.2 Hz, 2H), 6.14 (d, J = 2.2 Hz, 2H), 6.09 (t, J = 2.2 Hz, 1H), 6.03 (t, J = 2.2 Hz, 1H), 4.44 (t, J = 8.7 Hz, 1H), 3.16-3.02 (m, 2H), 2.77-2-60 (m, 4H). ^{13}C NMR (150 MHz, CD_3OD) δ (ppm): 158.8 (× 2C), 158.4 (× 2C), 155.6, 153.4, 148.2, 145.4, 133.3, 132.9, 131.8, 130.6 (× 2), 129.0, 127.2, 115.5, 115.2 (× 2C), 107.9 (× 2C),

107.8 (× 2C), 100.8, 100.7, 40.9, 39.2, 39.1, 37.8. Anal. calcd. for $C_{28}H_{26}O_6$: C, 73.35; H, 5.72. Found: C, 73.22; H, 5.73.
